# Use of the Rat Grimace Scale to Evaluate Visceral Pain in a Model of Chemotherapy-Induced Mucositis

**DOI:** 10.3390/ani9090678

**Published:** 2019-09-12

**Authors:** Rebecca P. George, Gordon S. Howarth, Alexandra L. Whittaker

**Affiliations:** 1 School of Animal and Veterinary Sciences, The University of Adelaide, Roseworthy Campus, Roseworthy, SA 5371, Australia; rebecca.george@adelaide.edu.au (R.P.G.); gordon.howarth@adelaide.edu.au (G.S.H.); 2 Department of Gastroenterology, Women’s and Children’s Hospital, North Adelaide, SA 5006, Australia

**Keywords:** rat grimace scale, chemotherapy-induced mucositis, disease activity index, open field test, opioids

## Abstract

**Simple Summary:**

Mucositis is a painful and often debilitating condition associated with cancer treatment. Management of associated symptoms is an important clinical consideration. Animal models are used in mucositis research to model the condition in humans in order to develop novel therapeutic agents to relieve symptoms. Previous animal studies have focused on disease severity and outcomes, but often failed to measure pain. The rat grimace scale (RGS) is a validated observational measure used to gauge pain levels experienced by rats. The aim of this study was to assess the rat grimace scale in a rat model of mucositis, and to examine whether changes in clinical signs and anxiety reflected the grimace responses recorded. We also aimed to determine whether the responses were pain-specific by administering potent opioid painkilling agents. In the present study rat grimace scores did not change significantly between treatments. Development of reliable pain assessment methods in animal models is urgently required to improve model relevance to human clinical practice, in addition to safeguarding animal welfare.

**Abstract:**

The rat grimace scale (RGS) is a measure of spontaneous pain that evaluates pain response. The ability to characterize pain through a non-invasive method has considerable utility for numerous animal models of disease, including mucositis, a painful, self-limiting side-effect of chemotherapy treatment. Preclinical studies investigating novel therapeutics for mucositis often focus on pathological outcomes and disease severity. These investigations fail to measure pain, in spite of reduction of pain being a key clinical therapeutic goal. This study assessed the utility of the RGS for pain assessment in a rat model of mucositis, and whether changes in disease activity index (DAI) and open field test (OFT) reflected the grimace responses recorded. Sixty tumor-bearing female Dark Agouti rats were injected with either saline or 5-Fluourouracil alone, or with co-administration of opioid analgesics. Whilst differences in DAI were observed between treatment groups, no difference in RGS scores or OFT were demonstrated. Significant increases in grimace scores were observed across time. However, whilst a statistically significant change may have been noted, the biological relevance is questionable in terms of practical usage, since an observer is only able to score whole numbers. Development of effective pain assessment methods in animal models is required to improve welfare, satisfy regulatory requirements, and increase translational validity of the model to human patients.

## 1. Introduction

Mucositis is a painful and debilitating condition arising in 40–60% of oncology patients treated with chemotherapeutic agents [1]. The condition results from a series of biological events initiated by the epithelial cell response to cytotoxic damage [2]. Pathological damage manifests as ulceration, inflammation and breakdown of the alimentary mucosal membranes. Mucositis affects the entire length of the gastrointestinal tract from mouth to the rectum [1]. Pain may arise directly due to the activation of nociceptors [3], or in response to other gastrointestinal events such as abdominal bloating and ulceration [4]. 

The frequency at which mucositis affects chemotherapy patients, and the serious nature of the symptoms it causes, render the mucositic condition to be the major dose-limiting factor in cancer treatment regimens [1]. Consequently, a range of research programs are directed at further elucidating the mechanisms of mucositis pathogenesis, and development of novel therapeutic agents for symptomatic relief of symptoms [5,6]. Many of these programs of research utilize animal models.

Legislation governing the use of animals in research frequently requires severity assessment of procedures performed on animals and amelioration of pain by analgesic administration [7]. However, objective, pain-specific parameters for severity assessment in rodents are lacking, and those that exist have not been widely validated. This has significant impacts on the ability to accurately apply humane endpoints and provide analgesic intervention. Further, since mucositis is a self-limiting condition, with symptoms self-resolving once cancer therapy ends, reduction of patient pain is a key goal of therapy [2]. Similarly, reduction of pain or other clinical signs is the primary aim in rodent models evaluating novel therapeutics. In clinical practice, patients are frequently prescribed potent opioid analgesics such as fentanyl and morphine to manage mucositis pain [8]. By extrapolation it might be assumed that rats with mucositis experience pain, and that this pain can be alleviated with opioid analgesics. Therefore, the use of opioid analgesics in this model may aid in validation of pain specific measures. The identification of a reliable measure of pain would considerably improve the translational validity of rodent models of mucositis. 

The rat grimace scale (RGS) is a measure of spontaneous pain that has been considered to evaluate the pain response [9]. The scale comprises four facial action units; orbital tightening, nose/cheek flattening, ear changes and whisker change. The RGS is well-studied, and validated, for the assessment of acute pain, such as that produced post-surgically [10,11]. However, in the face of visceral pain, such as intestinal mucositis, the RGS is less studied and the literature available is generally conflicting as to the validity of this scoring method [12]. Instead, gastrointestinal models tend to employ typical clinical scoring schemes arguably more indicative of general health, rather than specific to pain [5,13]. The disease activity index (DAI) evaluates disease severity based on general clinical observations; overall appearance of the animals, bodyweight loss, rectal bleeding and stool consistency. While an association between clinical signs and pain has been demonstrated in gastrointestinal disorders in human clinical practice, this has not been validated in rodent models of mucositis [3,14]. The open field test is a well validated measure of anxiety in rodent models. Although it does not measure pain, previous studies have shown that visceral pain and anxiety are often interrelated [15]. Therefore, the open field test has the potential to be an adjunctive tool for measuring affective state and associated pain. The aim of the current study was to assess the utility of the RGS for pain assessment in female Dark Agouti tumor-bearing (mammary adenocarcinoma) rats with chemotherapy-induced mucositis. The Dark Agouti rat mammary adenocarcinoma model (DAMA) was specifically chosen as it is extensively used in research investigating mucositis in rats [16]. The Opioid analgesics were administered to determine if the responses were pain-specific. A secondary aim was to examine whether changes in other measures of affective state, such as the DAI and the open field test, reflected the grimace responses recorded. 

## 2. Materials and Methods

### 2.1. Animals and Experimental Design

**Figure 1:**
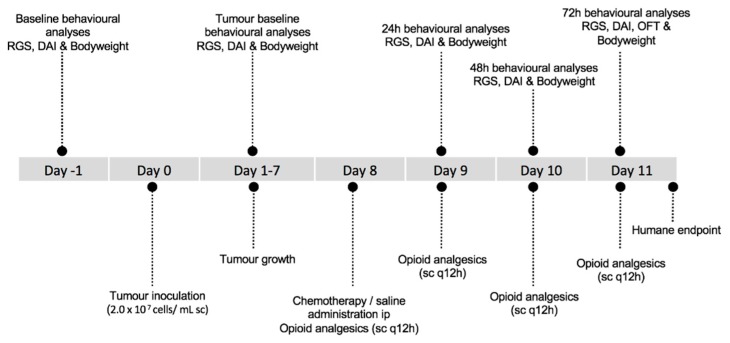
Overview of the experimental timeline for treatment and behavioral analyses. Abbreviations: RGS—rat grimace scale, DAI—disease activity index, OFT—open field test.

Female Dark Agouti rats (DA/Arc; n = 60) were acquired from a Specific-Pathogen Free production facility, Animal Resources Centre (ARC) (Perth, Western Australia). The supply facility undertakes a quarterly health screening program, covering a range of bacterial, viral and parasitic organisms, for which the colony screened negative. Rats were group-housed in groups of 5 in standard open-top polycarbonate rat cages (50 cm × 31 cm; Tecniplast, NSW, Australia) with ad libitum access to potable reverse osmosis treated water, a standard rat chow (Specialty Feeds, Glenn Forest, WA, Australia), and provided shredded paper as enrichment. Lights were set to a 12-h light-dark cycle and animal facility room temperature was maintained between 21 °C and 23 °C. Rats were given a seven-day acclimatization period. During this period rats were handled daily and habituated to the grimace observation chamber and open field arena. Following baseline behavioral analysis, rats, weighing approximately 140 g, were anaesthetized with isoflurane (induction 4%, maintenance 1–2% to effect, in O_2_). Isoflurane was selected as it provides rapid induction and recovery. Once anaesthetized, animals were injected with 0.2 mL (2.0 × 10^7^ cells/mL) of breast cancer inoculum into the right flank as described by Gibson et al., 2002; day 0 [17]. Tumors were allowed to grow for seven days. Rats were given wet food and clinical scoring was performed daily. On day 8, a random number generator was used to assign rats to five groups (n = 12) into which the animals were allocated. The experimental groups were: (1) saline injection (ip) + saline (sc q 12 h); (2) 5-Fluourouracil (5-FU) (150 mg/kg; ip) + saline (sc q 12 h); (3) 5-FU (150 mg/kg; ip) + morphine (3.33 mg/kg; q 12 h sc); (4) 5-FU (150 mg/kg; ip) + fentanyl (10 μg/kg; q 12 h sc); and (5) 5-FU (150 mg/kg; ip) + oxycodone (3 mg/kg; q 12 h sc). When determined by group allocation, saline was administered in an equivalent volume to 5-FU. Opioid analgesics and equivalent volume of saline were administered by subcutaneous injection at 12 hourly intervals for the remainder of the study (a total of 6 doses) (Figure 1). Dosages of all agents administered, and dosing schedule, were based on previous literature [18,19]. Sample sizes were chosen using RGS data from a previous publication [20], with an alpha value of 0.05, power 0.8, and mean difference of 0.3, indicating a requirement for 12 animals per treatment group. The study was carried out in 6 balanced replications over a three-month time period. Rats were humanely euthanized via CO_2_ asphyxiation 72 h post chemotherapy and saline administration. Rats were placed individually in a CO_2_ chamber, with a gradual fill rate of 20% of chamber volume/min. Death was confirmed by lack of corneal reflex and cessation of heartbeat. 

Animal housing and experimental protocols were approved by the Animal Ethics Committee of The University of Adelaide and conducted in accordance with the provisions of the Australian Code for the Care and Use of Animals for Scientific Purposes [21]. This paper was written in accordance with Animal Research: Reporting in vivo experiments: The ARRIVE guidelines [22]. This study was conducted as part of a larger experiment evaluating the influence of chemotherapy-induced mucositis and opioid palliation on the development of chemotherapy-induced cognitive impairment. 

### 2.2. Facial Image Analysis

Grimace scoring was performed at the same time of day for each animal during the light phase of the circadian cycle, at 5 time-points: 24 h prior to injection of tumor inoculum, 24 h prior to 5-FU or saline administration, and 24 h, 48 h and 72 h post-5-FU or saline injection. The latter three time points were selected to include an observation point 2 h following analgesic injection. Rats were removed from the home cage and placed in a 34 cm × 20 cm × 21 cm clear chamber. Two video cameras (Panasonic Video Camera HC-V180, Selangor, Malaysia) were placed perpendicularly on the outside sides of the chamber, with the two remaining sides and base being covered with white paper to enhance the clarity of the recordings. Rats were video recorded for a five minute-duration and then placed back into the home cage. All facial grimace recordings were performed in a quiet environment, in white light (150 lux) with no experimenter present. For image extraction, at each time-point a still image was selected and retrieved every 6 s using a video to JPG converter software (Free Studio v. 5.0.101 build: 201, United Kingdom). This occurred until a total of fifty images was obtained. Ten images of the rat’s face were then randomly selected from the still images using a random number generator. Images were only selected when photos showed rats directly facing the camera, and were then cropped to show the face alone. In the scenario where an image did not show the rat directly facing the camera, a new random number was generated. Three final images for scoring were then selected using a random number generator. All images were selected by an operator who was blinded to the group and time-point. The images were placed into a pre-designed excel spreadsheet for scoring and assigned a random number code. Scoring was performed by a treatment-blinded experienced coder using the method described by Sotocinal et al. 2011 [9]. Each image was scored based on four action units: orbital tightening, nose/cheek flattening, ear changes and whisker change. A score from 0–2 (0 = not present, 1 = moderate, 2 = severe) was assigned to each facial unit. The four action unit scores were summed to produce the total score and a mean of the scores for all three images obtained. 

### 2.3. Disease Activity Index

Disease activity index (DAI), [5] was recorded at 5 time-points; 24 h prior to tumor inoculum (baseline), 24 h prior to 5-FU and saline administration (tumor baseline), and 24 h, 48 h and 72 h post-injection. DAI scoring was conducted by a blinded observer, 2 h following analgesic injection prior to facial image analysis. DAI was measured on a scale of 0–3 severity (scored from 0 normal to 3 increasing in morbidity) per parameter based on overall appearance of the animals [23], bodyweight loss, rectal bleeding and stool consistency (Table 1).

### 2.4. Open Field Test

Anxiety level and locomotor activity were measured using the open field test (OFT) 72 h post 5-FU or saline administration. The OFT was conducted 1 h following facial image analysis. The OFT consisted of an enclosed, open field arena (1000 cm × 1000 cm × 1000 cm) in a brightly lit room, with a video camera (Logitech HD Webcam C525, Lausanne, Switzerland) suspended above the arena. The test commenced when a rat was placed in the center of the arena. After five minutes the rat was removed and placed back into the home cage. Each test was video recorded for the five minute period. Between each test the arena was cleaned using 70% ethanol. Analysis was performed retrospectively by a blinded observer using ANY-maze™ software (Stoelting Co.,Wood Dale, IL, USA). Two zones, peripheral and center, were superimposed, and the time spent, distance travelled and number of zone crosses for each zone were analysed. 

### 2.5. Statistical Analysis

Statistical analyses were conducted using Megastat Excel Add-In (version 10.3 Release 3.1.6 Mac, McGraw-Hill Higher Education, New York, NY, USA) and SPSS software (SPSS Inc., Chicago, IL, USA). Data were tested for normality and homogeneity of variance using the Shapiro–Wilk test. One animal was excluded from the study and subsequent analyses due to unrelated health issues, resulting in an n = 11 for the saline control group. RGS and DAI data between groups at each time point were analysed non-parametrically using a Kruskal-Wallis test with a *post-hoc* Mann Whitney U-test. The Friedman test with a *post-hoc* Mann Whitney U-test was applied to determine within group significances across time for both RGS and DAI data. Bonferroni correction was applied where appropriate to account for multiple comparisons. A repeated measures analysis of variance (ANOVA) with Tukey’s post hoc test was used to analyze body weight and open field data. Significance was determined at *p* < 0.05.

## 3. Results

### 3.1. Facial Image Analysis

**Figure 2:**
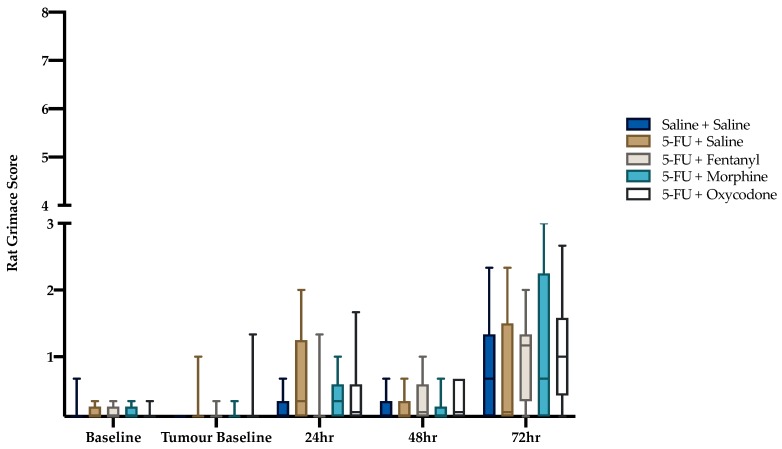
Box plot of rat grimace score. Maximum obtainable score was 8. Statistical comparison within group across time not shown (refer to Table 2). Saline + Saline (n = 11), 5-FU + Saline (n = 12), 5-FU + Fentanyl (n = 12), 5-FU + Morphine (n =12) and 5-FU + Oxycodone (n = 12) at all time-points.

There were no significant differences in RGS between treatment groups at any individual time-points (baseline *p* = 0.98; tumor baseline *p* = 0.68; 24 h *p* = 0.20; 48 h *p* = 0.57; 72 h *p* = 0.58) (Figure 2). Friedman analysis determined that the RGS scores were significantly higher post treatment (χ^2^(4) = 68.7, *p* < 0.001). Post-hoc Mann Whitney U-test determined that grimace scores were significantly increased for rats in all treatment groups at the 72 h time-point compared to tumor baseline (*p* < 0.05) (Table 2). 

### 3.2. Disease Activity Index and Body Weight

**Figure 3:**
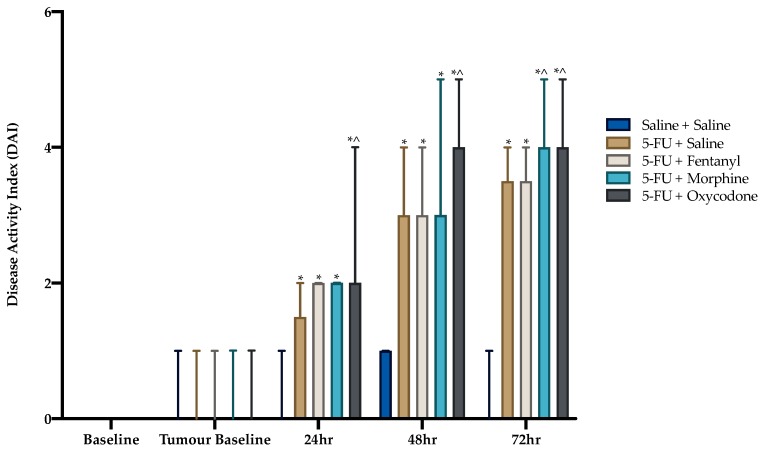
Disease activity index scores. Data expressed as median with range. Symbols denote significance obtained using a Kruskal-Wallis test with a *post-hoc* Mann Whitney U-test, between groups, at each time point. * *p* < 0.05 compared to saline. ^ indicates *p* < 0.05 compared to 5-FU alone. Statistical comparison within group across time not shown (refer to Table 2). Saline + Saline (n = 11), 5-FU + Saline (n = 12), 5-FU + Fentanyl (n = 12), 5-FU + Morphine (n = 12) and 5-FU + Oxycodone (n = 12) at all time-points.

Disease activity index scores increased significantly over time (*p* < 0.05). This increase was potentiated at 24 h, 48 h and 72 h compared to tumor baseline (*p* < 0.001, Table 2). There were no significant differences in DAI between treatment groups prior to 5-FU administration (*p* > 0.05). Administration of 5-FU significantly increased DAI within each time-point compared to saline control (Figure 3). The analgesic agents morphine and oxycodone increased DAI compared to 5-FU alone at later time-points (refer to Figure 3). 

**Figure 4:**
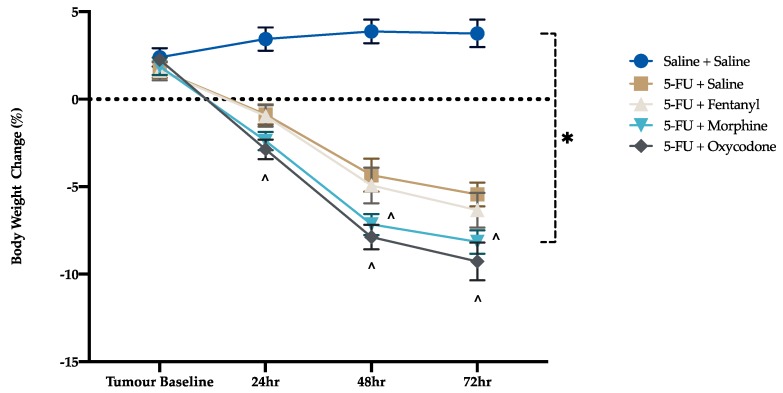
Body weight change. Data presented as mean % change in body weight ± standard error of the mean. * *p* < 0.05 compared to saline, ^ *p* < 0.05 compared to 5-FU. Saline + Saline (n = 11), 5-FU + Saline (n = 12), 5-FU + Fentanyl (n = 12), 5-FU + Morphine (n = 12) and 5-FU + Oxycodone (n = 12) at all time-points.

There were no significant differences in body weight between groups prior to chemotherapy injection. After chemotherapy administration there was a significant reduction in body weight at 24, 48 and 72 h in comparison to tumor baseline (F (4, 270) = 414, *p* < 0.001) (Figure 4). Body weight was further decreased for animals that received 5-FU in combination with analgesics compared to saline-injected rats. Morphine and oxycodone potentiated this weight loss compared to the 5-FU control group (24 h oxycodone alone, *p* = 0.044, 48 h; *p* = 0.008, *p* < 0.001, 72 h; *p* = 0.01, *p* < 0.001, respectively). 

### 3.3. Open Field Test

**Figure 5:**
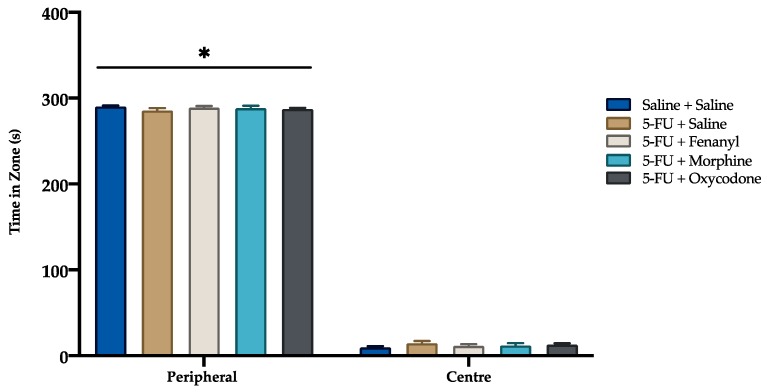
Time spent in center and peripheral zones of the open field test. Data presented as mean ± SEM. * *p* < 0.05 compared to center zone. Saline + Saline (n = 11), 5-FU + Saline (n = 12), 5-FU + Fentanyl (n = 12), 5-FU + Morphine (n = 12) and 5-FU + Oxycodone (n = 12).

There were no significant differences between treatment groups in the time spent in the centre or peripheral zones (*p* = 0.638, *p* = 0.650; respectively) (Figure 5). However, all rats, irrespective of treatment group, spent more time in the peripheral zone compared to the center zone (*p* < 0.05). No significant difference was detected between treatment groups in the distance travelled (centre, *p* = 0.415; peripheral, *p* = 0.494) and number of zone crosses (entries and exists) (centre, *p* = 0468, *p* = 0.470; peripheral, *p* = 0.540, *p* = 0.536; respectively), for both centre and peripheral zones. 

## 4. Discussion

In the current study we aimed to determine, firstly, whether rats with implanted tumors and experiencing mucositis displayed pain as determined through expression of a ‘pain face’, and secondly, whether this pain could be ameliorated through the use of potent opioid analgesics, hence validating the RGS as a pain measure in this model. A further aim was to examine whether the condition of the rats and accompanying analgesic treatment led to a change in affective state as evaluated through a standard general clinical scoring protocol and an established test of anxiety. No difference in pain scores was demonstrated between groups at any individual time points. An important observation was that, while a significant increase in RGS was observed from baseline to 72 h post 5-FU and saline administration for all groups, the scores were consistently low, and frequently below the threshold able to be scored by an unbiased observer using a point system. 

Our study findings imply that either: (1) rats with tumors/mucositis experienced minimal pain; or (2) the RGS lacked the sensitivity to successfully discriminate pain in this model. In consideration of the DAI scores, it becomes clear, however, that animals were experiencing some adverse welfare impact as evidenced by the increased scores, which reached approximately 50% of the total score possible at the later time-point. This scoring method is not unique to assessment of pain. Given the current findings of weight loss and changes in appearance in these rats, and previous studies demonstrating that other behavioral indicators of pain were exhibited at the time points evaluated [12], it is probable that the RGS is not a valid indicator of pain in this model. These results may have more general application to other gastrointestinal models of disease such as IBD and colitis where an acute visceral pain insult is anticipated, although there is some evidence to the contrary [24]. 

It is feasible that in the current study there was a confounding effect of the analgesic agent on assessment of the RGS score. For example, opioid agents may have produced a sedative effect thus influencing the facial action units. This may explain the significant RGS score exhibited in the morphine group at the 24 h time-point compared to tumor baseline, although this was not observed with other opioid agents. However, no change in spontaneous locomotor activity was measured in the open field apparatus, and based on previous literature this renders a sedative effect unlikely [25]. Furthermore, previous studies have disputed an influence of opioids on grimace tests [11,26]. The selected dosing regimens and time period for behavioral analysis may be factors in the minimal RGS response observed to the opioid treatments. Additionally, a significant increase in RGS score was observed from tumor baseline to 72 h in the saline control group. This elevation in RGS score may have been influenced by the pain caused by tumor burden, since this group had the largest tumors, being untreated. At 72 h there was also a trend towards significance in the DAI scores for these animals. However, we again urge caution in interpretation of these findings given the low scores, and the lack of sensitivity of the RGS over small changes, due to ordinal scoring.

Alternatively, the minimal RGS change exhibited may have arisen as a result of the time points chosen for data analysis. The peak of histological damage in 5FU-induced mucositis typically occurs at 48–72 h following injection [5,27], and thus pain was expected to be maximal at this point. However, it is feasible that histological damage and maximal pain experience may not be coincident. Alternatively, the ‘pain face’ may be inhibited by animals in longer term pain, due to the need to avoid predator attention [9]. The majority of studies to date have demonstrated declines in RGS following an initial peak in response to a painful insult by 6–8 h following the noxious stimulation [28,29,30]. However, previous studies involved a surgical stimulus [28,29,30], or inflammatory pain model [28], hence pain duration in the current study may have been reduced in comparison to the visceral pain sensation of mucositis. Contrarily, an assumed chronic pain insult caused by tooth movement caused a grimace response in rats for several days post-pain insult [31], as did the presence of colitis [14]. It is noteworthy that in the current study, grimace values were a small fraction of the maximum obtainable score (i.e., 8); often scoring under one. Therefore, while a statistically significant change may have been noted, the biological relevance is questionable in terms of practical usage, since an observer is only able to score whole numbers. 

It is plausible that the chosen observation period for grimace analysis and strain of rat used in the current study may have contributed to the RGS results obtained. Animals in this study were video recorded for a shorter duration compared to previous studies [9,14]. Exploration of the relatively new environment may have masked grimace response, with animals not having time to habituate to the chamber. However, changes in rat grimace score following recovery from exposure to either isoflurane or air have been reported using an identical scoring period to that used in the current study (five minutes) [32]. Furthermore, the rat strain used may have impacted on the ability to accurately score facial features. Previous research has implied that detection of grimace facial features in dark-colored animals is impaired [33,34]. This may however be as a result of poor achievement of background contrast, rather than the pigmentation of the animal per se. We tried to minimize this factor by using a white background when performing grimace scoring. Nonetheless, this could be a limitation of the study. Furthermore, our findings concur with previous results in a 5-FU induced mucositis model in white Sprague-Dawley rats, where mucositis did not elevate RGS in a longitudinal study design in which rats acted as their own controls [12].

The DAI has been widely used in models of mucositis to assess presence of the mucositic condition, in addition to severity, and is generally the sole assessment method utilized for making decisions on humane endpoint implementation, as prescribed by Animal Ethics Committees. The DAI findings in this study mirror those described in other mucositis studies with a progression in clinical signs until 72 h post-chemotherapy injection. However, the DAI is not specific to pain and therefore may not be useful in guiding when analgesic intervention is required. Further, it is apparent that the analgesic agents, morphine and oxycodone, actually increased the DAI score, thus challenging its interpretation. Closer inspection of the individual scores contributing to total DAI reveals that score is heavily influenced by weight loss, which increased over time in concert with histological disease progression, as described in previous studies. Since morphine and oxycodone potentiated weight loss, the DAI scores for these groups were relatively higher. If we assume that oxycodone is controlling pain, it is assumed that this exacerbation of bodyweight loss was brought about either through reduced appetite as a result of nausea, or as a result of a sedative effect rendering a larger proportion of the time budget being spent engaged in sleep. These are well recognized side effects of opioid administration [35,36]. This observation is consistent with previous studies, which have shown that the opioid analgesic, buprenorphine, similarly exacerbated bodyweight loss [27,35,37]. This lack of sensitivity of the DAI poses an issue when implementing humane endpoints. If strict point-score cut-offs are applied without the input of clinical judgement, animals may be withdrawn from a study as a result of analgesic side-effects such as weight loss, which influenced DAI. These side effects may not in themselves negatively impact on affective state. This poses an ethical issue in terms of animal wastage. 

## 5. Conclusions

There is a genuine need to develop and validate objective pain assessment tools in animal models of gastrointestinal disease such as the mucositis model described here. This will not only improve animal welfare and satisfy legislative requirements, but improve the translational validity of the models. Our data imply that the RGS fails to meet this need in a rat model of chemotherapy-induced mucositis and alternative pain assessment strategies are required. Alternative methods of emotional affect should be investigated, to include activities of daily living, such as burrowing and nest building, measures of affective biasing, or the use of running wheel activity. The conditioned place preference test might also be employed to examine preference for a substance assumed to provide an ameliorating effect [38]. Taking our findings, and those of others, into consideration, we also urge caution in conflating statistical significance with clinical significance when it comes to validating tools for practical implementation. Furthermore, given an apparent lack of sensitivity of traditional DAI scoring to side-effects of opioid analgesics, the comparative efficacy of opioid analgesics in these models cannot be established.

## Figures and Tables

**Table 1:** Disease activity index scoring criteria.

Overall Appearance *	Weight Loss (%)	Stool Consistency	Rectal Bleeding	Score
Normal	No weight loss	Normal Stools	No observable blood	0
Mild	0–5%	Loose	Blood spots in faeces	1
Moderate	5–10%	Mild diarrhoea	Clear evidence of blood in faeces	2
Severe	>10%	Overt diarrhoea	Gross bleeding	3

* Overall appearance was determined based on criteria; dull or ruffled coat, hunched, pale or sunken eyes, change in behavior, reduced food or water intake, dehydration, squealing when handled, reluctant to move.

**Table 2:** Statistical comparison within group across time for RGS and DAI.

	RGS	DAI
*Saline + Saline*	*P* Value	*P* Value
Tumour baseline vs. 24hr	0.28	0.54
Tumour baseline vs. 48hr	0.07	0.07
Tumour baseline vs. 72hr	0.03 *	0.06
*5-FU + Saline*		
Tumour baseline vs. 24hr	0.06	<0.001 *
Tumour baseline vs. 48hr	0.53	<0.001 *
Tumour baseline vs. 72hr	0.03 *	<0.001 *
*5-FU + Fentanyl*		
Tumour baseline vs. 24hr	0.91	<0.001 *
Tumour baseline vs. 48hr	0.12	<0.001 *
Tumour baseline vs. 72hr	0.001 *	<0.001 *
*5-FU +Morphine*		
Tumour baseline vs. 24hr	0.03 *	<0.001 *
Tumour baseline vs. 48hr	0.47	<0.001 *
Tumour baseline vs. 72hr	0.01 *	<0.001 *
*5-FU + Oxycodone*		
Tumour baseline vs. 24hr	0.11	<0.001 *
Tumour baseline vs. 48hr	0.12	<0.001 *
Tumour baseline vs. 72hr	0.001 *	<0.001 *
